# First Evidence of an Important Organic Matter Trophic Pathway between Temperate Corals and Pelagic Microbial Communities

**DOI:** 10.1371/journal.pone.0139175

**Published:** 2015-10-14

**Authors:** J. A. Fonvielle, S. Reynaud, S. Jacquet, B. LeBerre, C. Ferrier-Pages

**Affiliations:** 1 Department of marine biology, Ecophysiology team, Centre Scientifique de Monaco, Monaco, Monaco; 2 UMR CARRTEL, INRA, Station d’Hydrobiologie Lacustre, Thonon-les-Bains, France; CAS, CHINA

## Abstract

Mucus, i.e., particulate and dissolved organic matter (POM, DOM) released by corals, acts as an important energy carrier in tropical ecosystems, but little is known on its ecological role in temperate environments. This study assessed POM and DOM production by the temperate coral *Cladocora caespitosa* under different environmental conditions. The subsequent enzymatic degradation, growth of prokaryotes and virus-like particles (VLPs) as well as changes in the structure of the prokaryotic communities were also monitored. *C*. *caespitosa* produced an important quantity of mucus, which varied according to the environmental conditions (from 37.8 to 67.75 nmol carbon h^-1^ cm^-2^), but remained higher or comparable to productions observed in tropical corals. It has an important nutritional value, as highlighted by the high content in dissolved nitrogen (50% to 90% of the organic matter released). Organic matter was rapidly degraded by prokaryotes’ enzymatic activities, and due to its nitrogen content, aminopeptidase activity was 500 fold higher than the α-glucosidase activity. Prokaryotes, as well as VLPs, presented a rapid growth in the mucus, with prokaryote production rates as high as 0.31 μg h^-1^ L^-1^. Changes in bacterial and archaeal communities were observed in the ageing mucus and between mucus and the water column, suggesting a clear impact of mucus on microorganism diversity. Overall, our results show that the organic matter released by temperate corals, such as *C*. *caespitosa*, which can form reef structures in the Mediterranean Sea, stimulates microbial activity and thereby functions as a significant carbon and nitrogen supplier to the microbial loop.

## Introduction

Tropical, temperate and deep-water corals produce a polysaccharide layer (i.e. mucus) around them, regularly released into seawater for vital processes such as feeding, sediment cleansing and defence against environmental stresses [1]. As all biofilms, mucus affects the nutrient fluxes across the host’s body surface, and has thus an important ecological role in modulating the interactions between corals and their environment ([2–4]). It also hosts a highly diverse microbial community, which in some cases, forms species-specific associations with corals [5–7]. Once released in seawater, mucus, mostly composed of organic matter (OM), acts as an energy and nutrient carrier since it efficiently traps living and dead particles, suspended in the water column, and increases their sedimentation and recycling into essential nutrients such as nitrogen, phosphorus and carbon [8, 9]. This process plays a key role in the functioning of reef ecosystems, supporting benthic communities, as well as bacterial growth [10–12]. Micro-organisms are the major players of this OM recycling [13–15], through their exoenzymatic activities [16]. Particulate organic matter (POM) is hydrolysed by a battery of enzymes into dissolved organic matter (DOM), itself reduced into smaller molecules and inorganic nutrients [17, 18], which are further incorporated into bacterial biomass, and then to higher trophic levels. The idea that heterotrophic bacteria form an important trophic link between DOM and higher trophic levels has been presented in the concept of the microbial loop [19], which is at the base of the marine food web. The flow of nutrients within an ecosystem depends on the rate at which nutrients are recycled within the microbial loop, since most of the primary production in many locations, including reefs, is based on recycled rather than on new nutrients [20, 21].

For all the reasons cited above, OM composition and production rates have been well studied in corals forming large reef constructions, such as the tropical [12, 22–24], and cold-water corals [25, 26]. Conversely, OM production has hardly been investigated in temperate corals [27], probably because they are mostly found as isolated colonies, with a limited impact for their surrounding environment. However, some species, such as the scleractinian coral *Cladocora caespitosa* can form large herms (reef constructions) in the Mediterranean Sea [28, 29], and its OM production can potentially function as a carrier of energy and nutrients to fuel coastal food webs. In addition, and contrary to tropical or cold-water corals, *C*. *caespitosa* experiences large fluctuations in irradiance, temperature and nutrients throughout an annual cycle [30, 31], which impact its physiology [30]. It is thus a good model to study the environmental-related changes in OM production and nutritional value. Another under-studied aspect, for all coral species, is the bacterial enzyme mediated-recycling of mucus. While studies have measured natural bacterial respiration rates on a mucus substrate [8, 32], only one assessed the enzymatic degradation of mucus by cultured bacteria, either pathogenic or commensal to corals [33]. Such activity is however the most appropriate to define the type and quantity of substrate available to the microbial communities and to assess the hydrolysis of the dissolved and particulate resources [16]. At last, viral and archaeal ecology and/or diversity in and out (near) this produced extracellular mucus have been poorly investigated.

The present study therefore investigates whether *C*. *caespitosa* releases significant quantities of dissolved and particulate organic carbon (DOC and POC, respectively) and nitrogen (DON and PON, respectively) into the surrounding waters and whether this process varies with light and temperature. The degradation rates of sugars and proteins *via* glucosidase and aminopeptidase activities were also assessed, as well as the subsequent growth of micro-organisms and viruses, and the structure of the prokaryotic community. Our aims were i) to test whether coral mucus production is important, dynamic, and varies according to the environmental conditions; ii) to target the microbial degradability of this mucus, i.e. the OM capacity to function as a nutrient carrier within temperate ecosystems; iii) to predict the possible implications for the associated microbial metabolism and food web interactions in the water column. The response to these questions will allow a better prediction of the ecological function of temperate corals OM, which is for the moment completely unknown.

## Material and Methods

### Experimental set up

Six colonies of the scleractinian temperate coral *Cladocora caespitosa* were collected by scuba diving during fall 2013 in La Spezia (44°03′N, 9°55′E, North Mediterranean Sea, Italy), where they are abundant, and transferred within the day to the Centre Scientifique de Monaco (http://www.centrescientifique.mc/en/). This field research was performed under annual research permit (unnumbered) issued by the Italian Ministry of Research to the Marine environmental Research Center of ENEA (National Agency for the new technology, energy, and sustained economic development, Italy), and transported to Monaco under the CSM permanent CITES MC003. They were stored in four 100 L tanks, continuously supplied with Mediterranean seawater at a rate of 20 L h^-1^, and exposed to a light intensity close to the natural irradiance, i.e. 75 μmol photon m^2^ s^-1^, with 12:12 (L:D) cycles provided by an HQI lamp (Tiger SM 230V-50Hz 250W ST, Faeber lighting system, Italy). They were allowed to recover for a month. After this recovery period, colonies were fragmented into 48 micro-colonies (8 per parent colony, *ca* 25% of the colonies) of 3 to 6 polyps (measuring between 0.83 ± 0.12 cm² and 1.77 ± 0.44 cm^2^ respectively), which were equally dispatched into the 4 tanks. As *C*. *caespitosa* is classified as an endangered species in the IUCN red list, we kept the colonies and grew them in the Centre Scientifique de Monaco for further studies. Temperature and/or irradiance were then changed over several weeks in each tank to created 4 conditions: a “winter condition” (15°C and 40 μmol photon m^2^ s^-1^; subsequently called T15L40), a “summer condition” (22°C and 200 μmol photon m^2^ s^-1^, called T22L200) and two intermediate conditions of 15°C and 200 μmol photon m^2^ s^-1^ (T15L200) and 22°C and 40 μmol photon m^2^ s^-1^ (T22L40). Temperature was continuously regulated at ± 0.2°C using heaters connected to controllers and filters were placed between the HQI lamps and the aquaria to reach the desired light intensities. Corals were fed twice a week with *Artemia salina* nauplii (Aqualiment, France) and were maintained up to 61 days under the above conditions before the following measurements were performed.

### Metabolic measurements

Calcification rates were measured on all micro-colonies (n = 12 per tank) using the buoyant weight technique [34]. Corals were weighed just before temperature and light were changed in each tank, and after 35 and 61 days. A skeletal density of 1.84 was used [35, 36] and data were expressed as mg CaCO_3_ deposited d^-1^ cm^-2^. Six micro-colonies per condition (one per parent colony) were also used after *ca*. 60 days to monitor the net photosynthesis (Pn) and dark respiration (R) according to Rodolfo-Metalpa et al [30]. They were frozen at the end of the experiment, for the determination of the chlorophyll and protein concentrations as well as the symbiont density, according to Rodolfo-Metalpa et al [30]. All measurements were normalised to the surface area of the polyps determined with a calliper as described in Rodolfo-Metalpa et al [30].

Measurements of total, dissolved and particulate organic carbon (TOC, DOC, POC), and nitrogen (TON, DON, PON) fluxes were performed after *ca*. 60 days according to the beaker incubation technique [24, 27]. All materials used were cleaned from organic matter in successive baths of 10% hydrochloric acid (during a night) and rinsed with distilled water, before being burned at 500°C during 6 h in an oven. Six micro-colonies per condition (one per parent colony) were incubated for 6 to 8 h in 250 mL beakers filled with 0.45 μm-filtered seawater, continuously stirred with a stirring bar. A water bath maintained the desired temperature in the beakers and light was provided by an overhead HQI lamp. Triplicate 10 mL seawater samples were drawn from each beaker using sterile syringes, pre-washed with few mL of samples, at the beginning (prior to the introduction of the corals), and at the end of the incubations (after careful removing of the corals from the beakers using sterile tweezers). Seawater samples were stored in pre-combusted glass vials at -20°C for the determination of TOC and TON fluxes. Another set of triplicate seawater samples (10 mL) was taken for the determination of DOC and DON fluxes. For this purpose, samples were filtered before storage onto 0.2 μm cellulose syringe filters (Sartorius Stedim Minisart, Sigma-Aldrich, USA), pre-washed with 6 mL of sample. Leakage of DOC/DON from the filter membranes was found to be insignificant, as quantified by preliminary experiments. POC and PON fluxes were deduced from the difference between the total and dissolved fractions. For analysis, DOC/POC samples were defrosted, acidified to a pH <2 by adding 2 μl of 2 mol l^-1^ HCl, and purged by bubbling O2 for 2 min to remove dissolved inorganic carbon. Samples were then analysed in triplicate using a TOC-L analyser (Shimadzu, Japan). The organic matter fluxes were normalised to the surface area of the micro-colonies and expressed as nmol cm^-2^ h^-1^. The analyser was calibrated each day with carbon and nitrogen standards (Hansell Laboratory, University of Miami, USA).

### Enzymatic activities

To assess the extracellular enzyme activities (EEA) in the mucus (TOC and TON) released, fluorescent substrate analogues were used according to the standard protocols [37]. Aminopeptidase and α-glucosidase activities were monitored using respectively the 4-Leucine-7-amido-4-methyl-coumarin hydrochloride substrate (Leu-MCA) and 4-methylumbelliferyl-α-D-glucopyranoside substrate (α-MUF) (Sigma-Aldrich, USA), which release a fluorescent compound under the action of extracellular enzymes. Correspondence between fluorescence value and quantity of substrate hydrolysed was obtained using amino 4-methylcoumarin (MCA) and 4-methylumbeliferone (MUF). Several concentrations (0, 10, 100, 150, 250, 300 and 500 μM) were tested in preliminary experiments (not shown) in order to get the maximal activity, which was obtained with a substrate concentration of 250 μM for both enzymes. Fluorescence was measured using 96 well plates of 300 μL and a spectrofluorometer (Xenius XM, Safas, Monaco), at wavelengths of 448–360 nm for MUF and 442–356 for MCA. pH was adjusted to 10.8 by adding Tris-HCl buffer, to obtain maximal fluorescence intensity.

Before EEA measurements, three 250 mL beakers per condition (n = 12 in total), filled with 0.45 μm filtered seawater, and containing 2 micro-colonies each (from different parent colonies), were incubated under the right temperature and light for 1 h as described above. Colonies were then removed from the beakers and each incubation medium, containing the mucus released, was divided into three sampling sets:

1. A first sampling set was used for EEA activity. For this purpose, three 1 mL sub-samples were incubated on a thermostatic shaking bath, for up to 240 h, in the dark and at the right temperature, in 15 mL disposable test tubes, where either α-MUF or Leu-MCA were added to a final concentration of 250 μM (final volume of 3 ml in each test tube). Enzymatic activities were measured after 1, 4, 8, 24, 48, 72, 96, 120, 148 and 240 hours as described above. Values were corrected against a blank without mucus. The amount of organic matter hydrolysed in one hour was then calculated taking into account the EEA (in nmol L^-1^ h^-1^) and the organic matter released (TOC or TON, in nmol L^-1^ h^-1^). This quantity was compared to the initial production of mucus to calculate the percentage of mucus hydrolysed, *i*.*e*. the mucus degradation rate. This rate allowed us to estimate OM turnover.
2. A second sampling set was used to assess the abundance of heterotrophic prokaryotes and virus-like particles. For this purpose, the remaining medium was incubated in the dark, during 240 hours. 4.8 mL sub-samples were taken after 1, 8, 24, 48, 72 and 96 hours and conserved in 5 ml cryogenic vials (Corning inc, USA) containing glutaraldehyde at a final concentration of 1%. Micro-organisms were fixed during 30 minutes in the dark at 4°C before being flash frozen in liquid nitrogen and stored at -80°C. Particle and cellular abundances were determined using a FACSCalibur flow cytometer (Becton Dickinson) as described by Jacquet et al [38]. To estimate bacterial biomass, we assumed an average value of 20 fg of carbon per cell as proposed previously [39, 40]. Maximal growth rates were calculated using the formula:
  $$\left(\left[T_{\text{f}}-{\text{T}}_{0}\right]\right)\;/\;\left({\text{T}}_{\text{f}}-{\text{T}}_{0}\right).$$
3. The last sampling set served to determine the bacterial and archaeal community structures of the mucus after 96 h incubation, using the Polymerase Chain Reaction (PCR) and Denaturing Gradient Gel Electrophoresis (DGGE). Cells were harvested from the remaining water onto 47 mm diameter, 0.2 μm pore size, polycarbonate white membrane filters (Nuclepore) after a pre-filtration step through 2 μm pore size polycarbonate membrane filters (Nuclepore) to eliminate small and large eukaryotes. The filters were then stored at -20°C until nucleic acid extraction, which was performed as described by Dorigo et al [41] using phenol-chloroform. Molecular weight distribution and purity of the DNA were assessed by 1% agarose gel electrophoresis and quantified by both visual comparisons with molecular weight markers in ethidium bromide stained agarose gels (rough estimate) and by optical density measurements using NanoDrop ND-1000 Spectrophotometer (Thermo Scientific). The extracted DNA was then stored at -80°C until PCR amplification.

For Bacteria, PCR reactions were carried out using the Eubacteria-specific primer 358-GC [42] and the universal primer 907rM [43] which amplify the variable V3 region of the 16S rRNA gene and yield a DNA fragment of ca. 550 bp. All PCR amplifications were carried out using about 30 ng of extracted DNA in a 25 μL reaction mix containing 10 X La buffer II (with Mg^2+^), 0.2 mM of each deoxynucleotide, 0.5 pmol of each primer, bovine serum albumin (Sigma, 0.5 mg ml^-1^ final concentration), and 0.625 U Takara LA Taq (Ozyme). PCR amplification consisted of an initial denaturation step of 94°C for 5 min, followed by 10 cycles of denaturation at 94°C for 1 min, annealing at 65°C (- 1°C/cycle) for 1 min, and extension at 72°C for 3 min, followed by 17 cycles at 94°C for 1 min, 55°C for 1 min, 72°C for 3 min and a final elongation step at 72°C for 5 min using a PTC100 thermocycler (MJ Research). Correct sizes (ca. 590 bp length) of PCR products were determined by 1% agarose gel electrophoresis with a DNA size standard (Low DNA Mass Ladder, GIBCO BRL). DGGE was carried out with a CBS system using a 6% (wet/vol) polyacrylamide gel (30–70% gradient). The gel was run at 120 V for 16 h at 60°C in TAE 1X. The gel was stained during 30 min using SYBRGold (1/5,000 final concentration) and bands were visualised under UV light and photographed using GelDoc (Biorad). PCR and DGGE targeting the Archaea were performed following the recently developed protocol of [44] for freshwater samples. We performed nested PCR for DGGE with primer sets 21F-958R (21F: 5’-TTC CGG TTG ATC CYG CCG GA-3’; 958R: 5’-YCC GGC GTT GAM TCC AAT T-3’) and Parch519-Arch915 (519: 5’-CAG CCG CCG CGG TAA-3’; 915:5’-GTG CTC CCC CGC CAA TTC CT-3’ with a 40 bp GC clamp attached to the 5’ end). PCR conditions were as described in Vissers *et al* [45]) but PCR amplification was performed in a total volume of 25 μL containing (in final concentration) 1X La PCR buffer II (Mg^2+^) associated with 0.2 mM of each deoxynucleotide, 0.5 pmol of each primer, 0.4 mg/mL of BSA, 1.25 U of Takara LA Taq (Takara Bio Inc.) and 25 ng of extracted DNA for the first PCR. The second PCR was performed using 0.25 μL of the product of the first PCR. Reactions were performed using the PTC-100 thermocycler (MJ research) with PCR amplification consisted of an initial denaturation step of 94°C for 5 min, followed by 25 cycles of denaturation at 94°C for 30 sec, annealing at 57°C for 40 sec, extension at 72°C for 40 sec, and a final elongation step at 72°C for 5 min. The correct sizes (*ca*. 440 bp length) of PCR products were determined as above. DGGE was carried out as described above but at 250 V for 5 h.

### Statistical analysis

Statistical analyses were performed with R software (R development core team http://r-project.org/). All data are mean ± standard deviation. A 2-way analysis of variance (ANOVA) was performed using multiple linear models with light and temperature as independent variables. Data were tested for variance homoscedasticity with a Bartlett test while normality was tested using Shapiro-Wilk test and log transformed when necessary. A post-hoc HSD Tukey test was performed when ANOVA showed a significant effect (p<0.05) of at least one factor. Differences between conditions were considered for p<0.05. Associations between measured parameters were analysed using linear regression, followed by F-test. Correlations were determined using Pearson’ test. A t-test was performed in order to determine if OM values were significantly different from 0 and considered significant for p<0.05. Comparative analysis of DGGE profiles, based on both presence and intensity of bands, was carried out with GelCompar II using a 2% tolerance for band’s separation. To visualize the relationship between communities throughout the sampling period, ordination of Bray-Curtis dissimilarities among normalized DGGE profiles was performed by hierarchical agglomerative clustering using unweight pair group method with arithmetic averages (UPGMA).

## Results

### Effects of light and temperature on the physiological parameters of *C*. *caespitosa*

**Fig 1:**
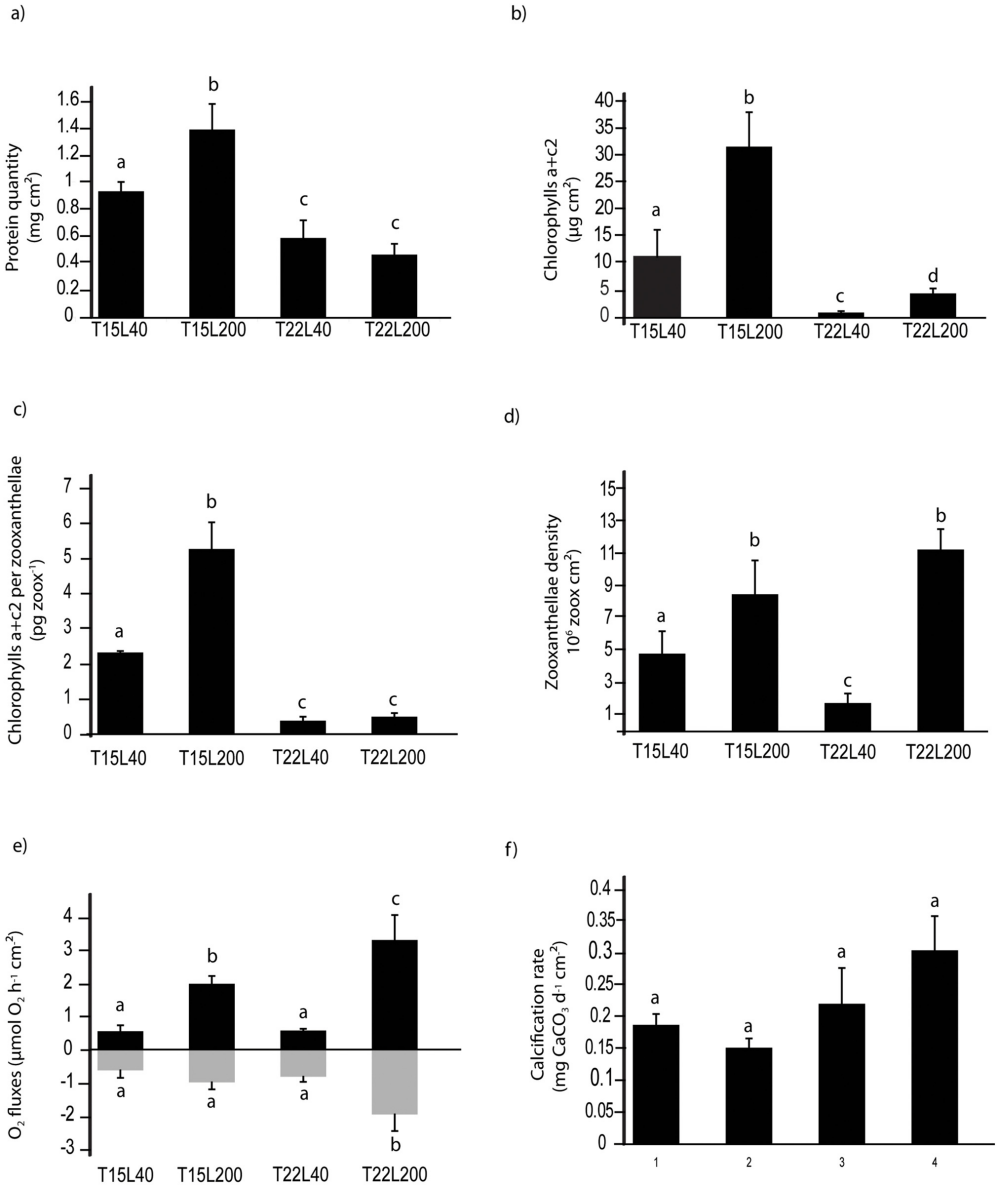
Changes in C. caespitosa physiological parameters at 15 or 22°C (T15 and T22 respectively) and 40 or 200 μmol photon cm^-2^ (L40 and L200 respectively) a) protein concentration per skeletal surface area b) chlorophyll concentration per surface area c) chlorophyll per symbiont cell d) symbiont density per surface area e) net photosynthesis (black) and dark respiration (grey) per surface area, and f) calcification rate. Data are mean (n = 6) ± s.d. (or s.e. for calcification rate), Different letters above each bar indicate significant differences.

Light and temperature had significant effects on the physiology of *C*. *caespitosa* (Table 1). Protein and chlorophyll (chl) concentrations per skeletal surface area (Fig 1a and 1b and Table 1) were significantly higher (p<0.01) at 15°C than at 22°C, and reached the highest value under high light (T15L200). Chlorophyll per symbiont cell (Fig 1c) was also significantly higher at 15°C (from 2.32 ± 0.06 μg cell^-1^ at T15L40 to 5.17 ± 1.15 μg cell^-1^ at T15L200) than at 22°C (from 0.38 ± 0.13 μg cell^-1^ at T22L200 to 0.49 ± 0.13 μg cell^-1^ at T22L40). Conversely to the above parameters, which were more influenced by temperature, symbiont density and rates of photosynthesis were mostly affected by light (Table 1). They were significantly higher under high light (Fig 1d and 1e, p < 0.05), with the highest value under high temperature (T22L200). There was a positive correlation between photosynthetic rates and symbiont density (r^2^ = 0.89, p = 0.05, data not shown). Respiration (R) was only higher (p<0.05) in T22L200. Calcification rates were not significantly impacted by either light or temperature (Fig 1f), although values tended to be higher in T22L200. Mean calcification rate for all conditions was 0.215 mg CaCO_3_ d^-1^ cm^-2^.

**Table 1:** Results of the two way analysis of variance with irradiance (L) and temperature (T) as independent variables performed with the R software for net photosynthesis (Pn), respiration (R), total, dissolved and particulate organic carbon (TOC, DOC, POC, respectively), and nitrogen (TON, DON, PON, respectively), the percentage of dissolved organic carbon (%DOC) or nitrogen (%DON), the abundance of prokaryotes and virus-like-particles, the virus to prokaryote ratio (VPR), and the degradation rate of total organic carbon and nitrogen (TOC and TON degradation rate, respectively). Bold p-value indicated p-value < 0.05 according to the degree of freedom (df) and the ratio between the sum of the mean square of the variance between groups and the sum of the mean square of the variance inside the groups (F-ratio).

	Factor	df	F-ratio	p-value		Factor	df	F-ratio	p-value
**Pn**	L	1	217.6	**2.21 10** ^**−16**^	**PON**	L	1	16.7	**3.50 10** ^**−4**^
	T	1	15.4	**2.13 10–4**		T	1	4.3	**0.047**
	L + T	1	14.2	**3.24 10–4**		L + T	1	8.9	**0.006**
**R**	L	1	116.2	**2.34 10** ^**−16**^	**% DON**	L	1	10.6	**0.004**
	T	1	69.6	**5.21 10** ^**−12**^		T	1	0.1	0.733
	L + T	1	30.9	**4.88 10** ^**−7**^		L + T	1	6.6	**0.018**
**TOC**	L	1	5.7	**0.023**	**TOC:TON**	L	1	8.2	**0.001**
	T	1	0.6	0.425		T	1	30.2	**2.64 10** ^**−5**^
	L + T	1	12.1	**0.002**		L + T	1	0.3	0.571
**DOC**	L	1	33.6	**3.58 10** ^**−6**^	**Prokaryotes abundance**	L	1	2.8	0.112
	T	1	6.9	**0.014**		T	1	44.2	**1.09 10** ^**−6**^
	L + T	1	4.4	**0.045**		L + T	1	0.7	0.411
**POC**	L	1	8.1	**0.009**	**VLPs abundance**	L	1	19.1	**2.24 10** ^**−4**^
	T	1	9.2	**0.005**		T	1	218.3	**3.15 10** ^**−13**^
	L + T	1	6.8	**0.015**		L + T	1	24.0	**5.99 10** ^**−5**^
**% DOC**	L	1	38.1	**4.93 10** ^**−6**^	**VPR**	L	1	19.1	**2.63 10** ^**−4**^
	T	1	6.3	**0.021**		T	1	1.3	0.262
	L + T	1	3.1	0,.102		L + T	1	3.6	0.073
**TON**	L	1	162.3	**4.72 10** ^**−11**^	**TOC degradation rate**	L	1	18.3	**4.46 10** ^**−4**^
	T	1	51.4	**6.07 10** ^**−7**^		T	1	5.1	**0.04**
	L + T	1	35.3	**8.15 10** ^**−6**^		L + T	1	151.2	**3.4 10** ^**−10**^
**DON**	L	1	32.8	**9.12 10** ^**−6**^	**TON degradation rate**	L	1	3.8	0.062
	T	1	65.6	**4.79 10** ^**−8**^		T	1	25.9	**5.59 10** ^**−5**^
	L + T	1	9.68	**0.005**		L + T	1	0.3	0.583

### Release rates of organic matter by *C*. *caespitosa*

**Fig 2:**
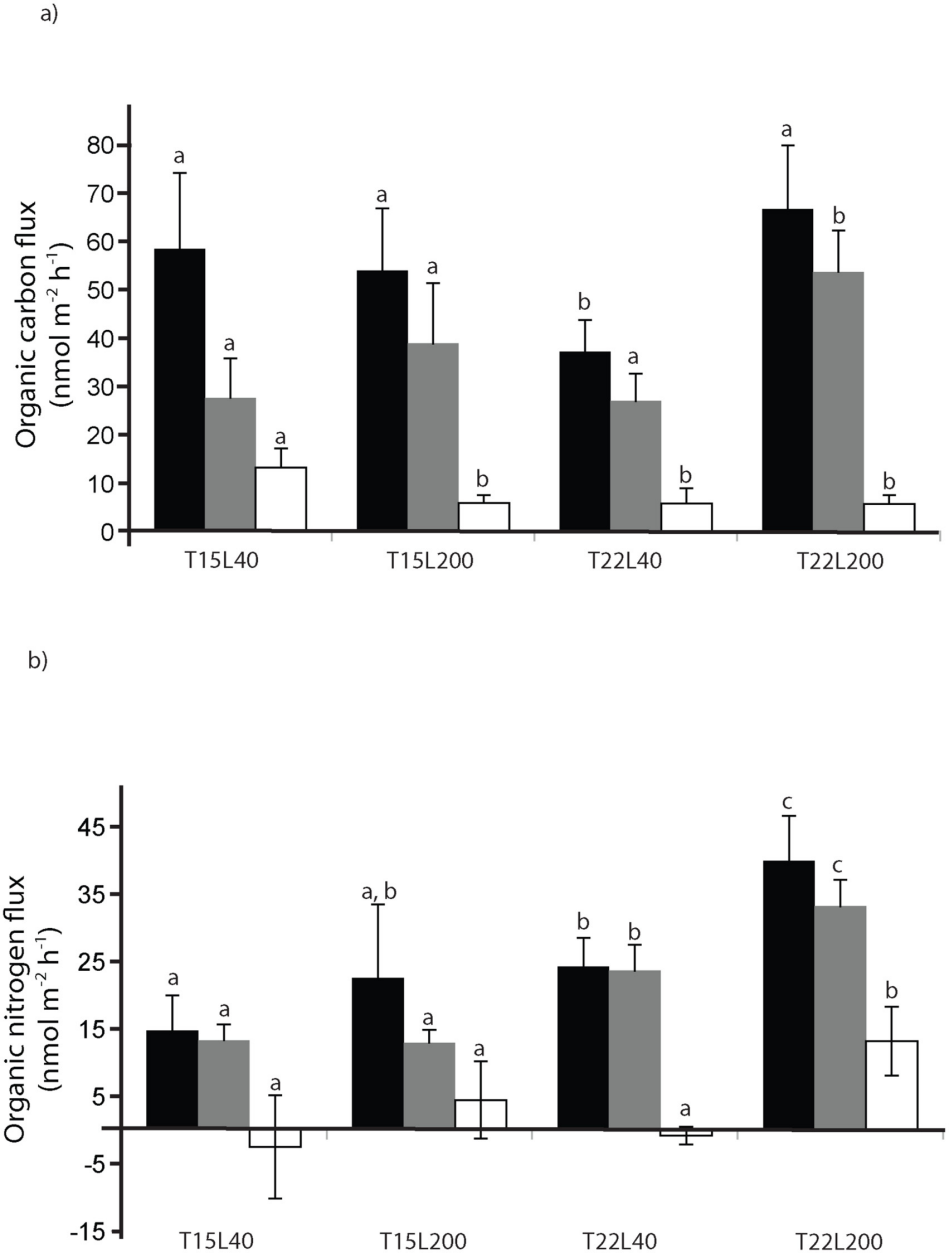
Fluxes of total (black) dissolved (grey) and particular (white) organic carbon (a) or nitrogen (b) produced by *C*. *caespitosa* at 15 or 22°C (T15 and T22 respectively) and 40 or 200 μmol photon m^-2^ s^-1^ (L40 and L200 respectively). Data are mean ± s.d. for n = 6 nubbins per condition. Different letters above each bar indicate significant differences.

The organic carbon and nitrogen fluxes in beakers containing coral colonies were significantly different (p<0.01) from the controls and positive, i.e. from the corals to the seawater, evidencing production of organic matter (Fig 2a and 2b). Among the major traits, corals excreted significantly (p<0.05) more dissolved organic carbon and nitrogen (DOC, DON) than particulate material (POC, PON) (Fig 2). Fluxes were impacted by light and temperature (Table 1). High light significantly increased DOC production (Fig 2a, Table 1), and also increased the proportion of PON *vs*. DON (p<0.05). High temperature significantly increased DON production (Fig 2b, Table 1). The TOC:TON ratio was higher (p<0.01) in T15L200 condition (4.14 ± 0.95) than in the other ones (from 1.45 ± 0.14 to 1.83 ± 0.54). Under high light, a significant positive correlation was found between TOC or TON release rate and photosynthesis rate (p = 0.01 and 0.03 respectively).

### Extracellular enzymatic activities (EEA)

Preliminary experiments were performed to determine the substrate concentration (α-MUF or Leu-MCA) that led to a maximal enzymatic activity. This maximum was obtained for both enzymes for a substrate concentration equal to 250 μM, which was the one used in the following experiments (results not shown).

**Fig 3:**
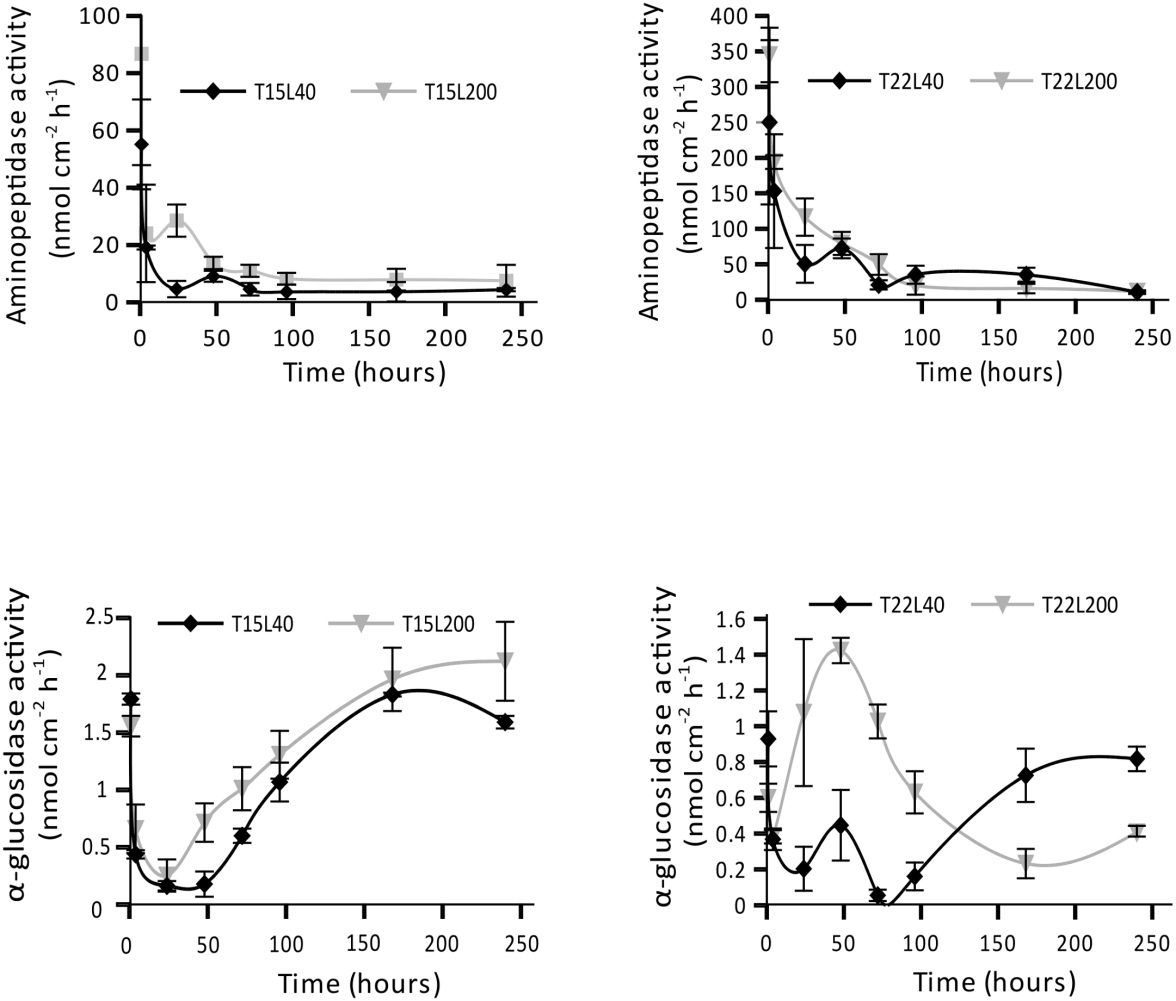
Variation in aminopeptidase (a, b) and glucosidase (c, d) activity with mucus aging at 15 or 22°C (T15 and T22 respectively) and 40 or 200 μmol photon m^-2^ s^-1^ (L40 and L200 respectively). Data are mean (n = 9) ± s.d. of triplicate analysis of three mucus collected using three different colonies.

**Fig 4:**
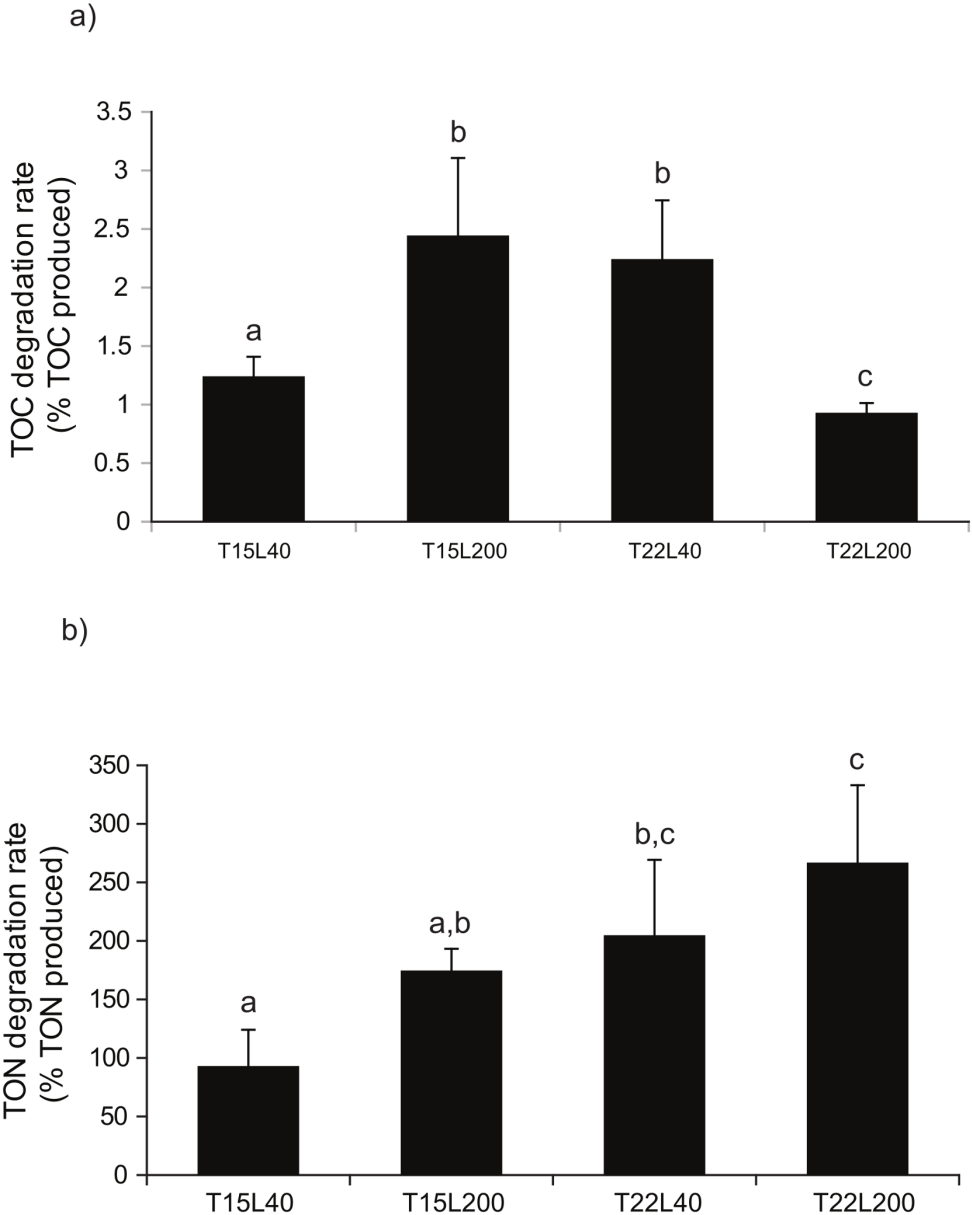
Organic carbon (a) and nitrogen (b) degradation rates at 15 or 22°C (T15 and T22) and 40 or 200 μmol photon m^-2^ s^-1^ (L40 and L200 respectively). Data are mean (n = 6) ± s.d., Different letters above each bar indicate significant differences.

EEA were assessed in freshly released mucus (TOC/TON) during 240 hours and were significantly different according to the light and temperature conditions (p<0.05). In addition, aminopeptidase activity was 100 to 200 times higher than the α-glucosidase activity. Temperature was the main parameter affecting the maximal aminopeptidase activity, which was *ca*. 4 times higher at 22°C than at 15°C and reached its maximal value at T22L200 (p<0.01, Fig 3a and 3b). This higher activity at 22°C was related to a higher TON release rate. The activity was also maximal at the beginning of the incubation for all conditions, then continuously decreased (Fig 3a and 3b), faster under low than high temperature. The plateau was thus reached after 48 h at 15°C *versus* 96 h at 22°C, at 8 nmol h^-1^ cm^-2^ and 15 nmol h^-1^ cm^-2^, respectively (Fig 3a and 3b). The maximal α-glucosidase was equivalent in all conditions, around 1.5–2 nmol h^-1^ cm^-2^ (Fig 3c and 3d). This activity was high right after coral OM was released, and then after 48 h in T22L200, and only after 240 h in the other conditions. According to the maximal amount of TOC released (67.65 nmol h^-1^ cm^-2^ in T22L200 condition, Fig 2a) and the glucosidase activity in this condition (1.5 nmole h^-1^ cm^-2^, Fig 3c and 3d), bacteria degraded 1.5% of the released TOC in one hour (Fig 4a). The maximal amount of TON produced by *C*. *caespitosa* was equal to 41.82 nmol h^-1^ cm^-2^ of coral. The aminopeptidase activities ranging between 100 and 350 nmol h^-1^ cm^-2^ of coral (Fig 3a and 3b) were much higher than TON production, suggesting than in all conditions, the entire TON produced was rapidly consumed (Fig 4b).

### Changes in micro-organism abundances in seawater

**Fig 5:**
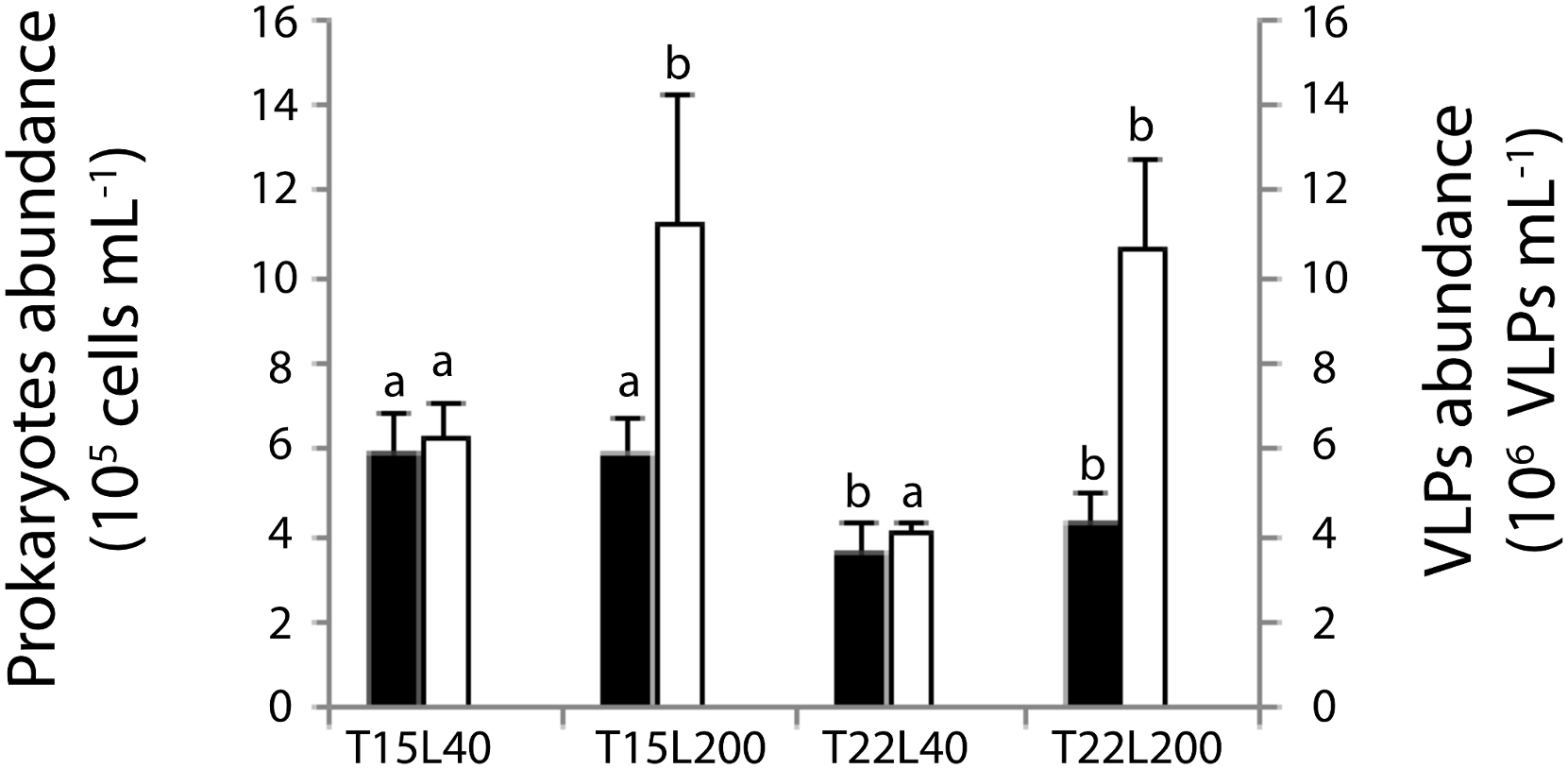
Variation in prokaryotes (black) and virus-like-particles (VLPs) abundance (grey) at 15 or 22°C (T15 and T22 respectively) and 40 or 200 μmol photon m^-2^ s^-1^ (L40 and L200 respectively). Data are mean (n = 9) ± s.d. of triplicate analysis of three mucus collected on each three paired-nubbins. Different letters above each bar indicate significant differences.

In freshly produced OM (T_0_), the abundance of heterotrophic prokaryotes was temperature-dependent, and significantly higher at 15°C than at 22°C (Fig 5a, Table 1). Conversely, the concentration of the virus-like particles (VLPs) was light-dependent, and significantly higher under high light, with the highest concentration in T15L200. The virus-to-prokaryote ratio (VPR) was also higher (p<0.01) in high light conditions (Table 1).

**Fig 6:**
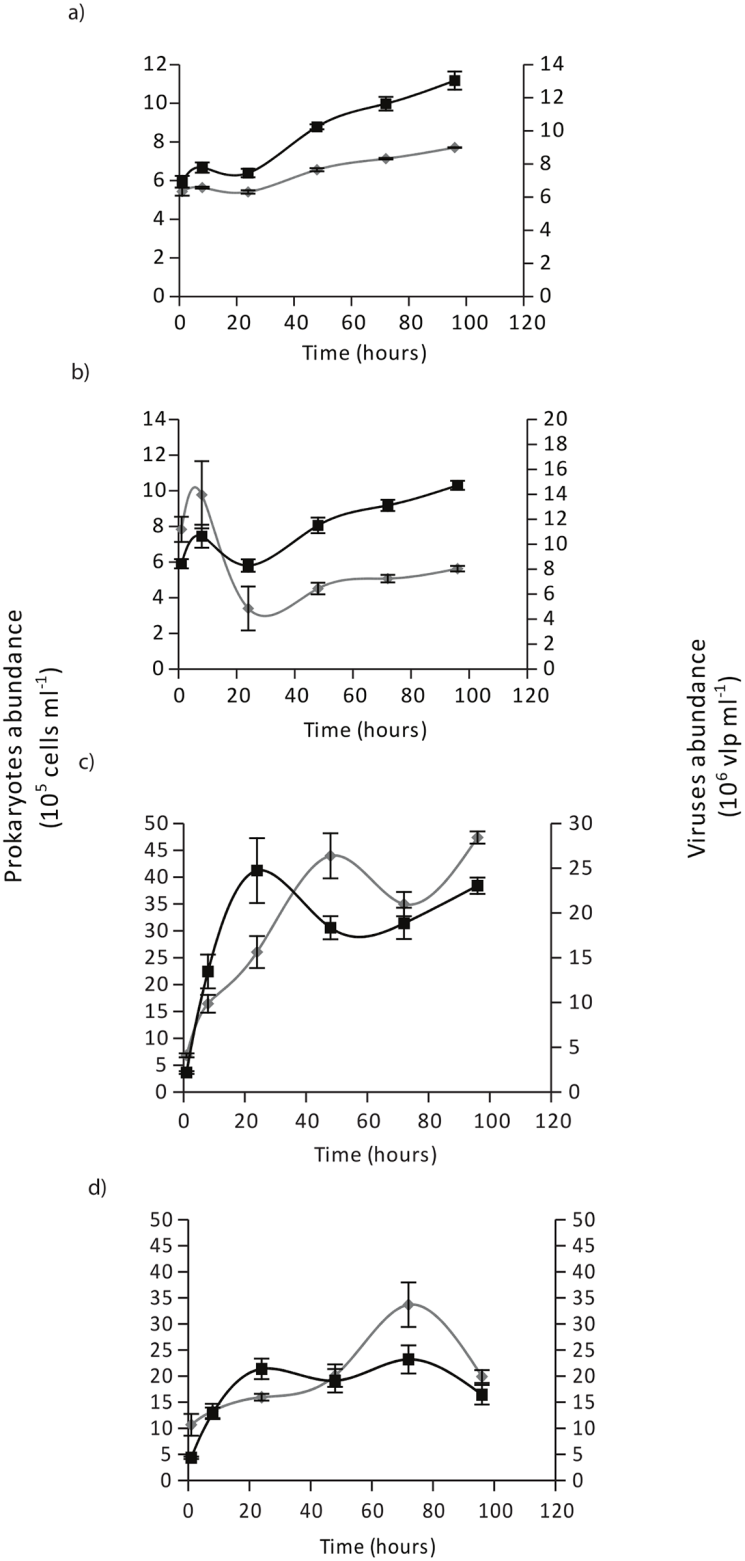
Changes in micro-organism abundances in the organic matter released by *C*. *caespitosa* during a 96 h incubation. Prokaryote (black squares) and virus-like-particles (VLPs) abundances (grey circle) were plotted together for T15L40 (a) T15L200 (b) T22L200 (c) and T22L40 (d) condition. Data are mean (n = 9) ± s.e. of three incubations of three different set.

OM degradation led to a rapid proliferation of prokaryotes and VLPs (Fig 6). At 15°C, prokaryotic abundance continuously increased during 96 h, and was multiplied by almost 2 during the incubation (from 6 to 11 x 10^5^ cells ml^-1^). The highest growth rates (μ) and production rates (P_B_) were measured at the end of the incubation (μ = 0.017 h^-1^, P_B_ = 0.013 μg h^-1^ L^-1^) without significant differences with the light regime. At 22°C, prokaryotic abundance was multiplied by 5 to 10 in 24 h. Growth and production rates were thus maximal at the beginning, of the incubation (μ = 0.1 h^-1^, P_B_ = 0.129 μg h^-1^ L^-1^ in T22L40 and 0.4 h^-1^, P_B_ = 0.310 μg h^-1^ L^-1^ in T22L200) and at least 10 times higher than at 15°C.

### DGGE analysis

**Fig 7:**
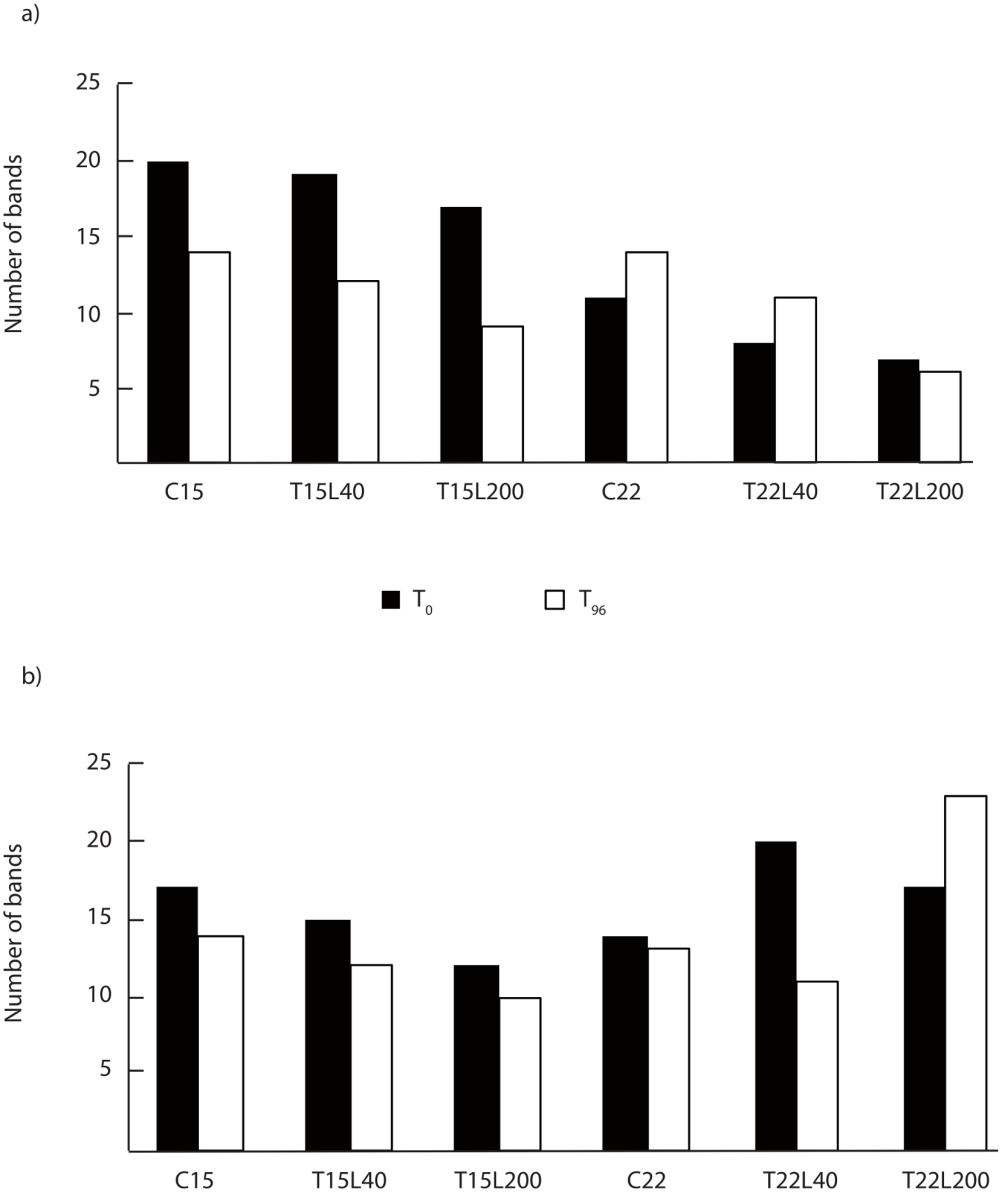
Number of bands observed after PCR DGGE analysis of the bacterial (a) and archaeal (b) community, at 15°C (C15) and 22°C (C22), and in the 4 experimental conditions (T15L40, T15L200, T22L40 and T22L200) just after the mucus was released (black) and after 96 h of incubation (white).

**Fig 8:**
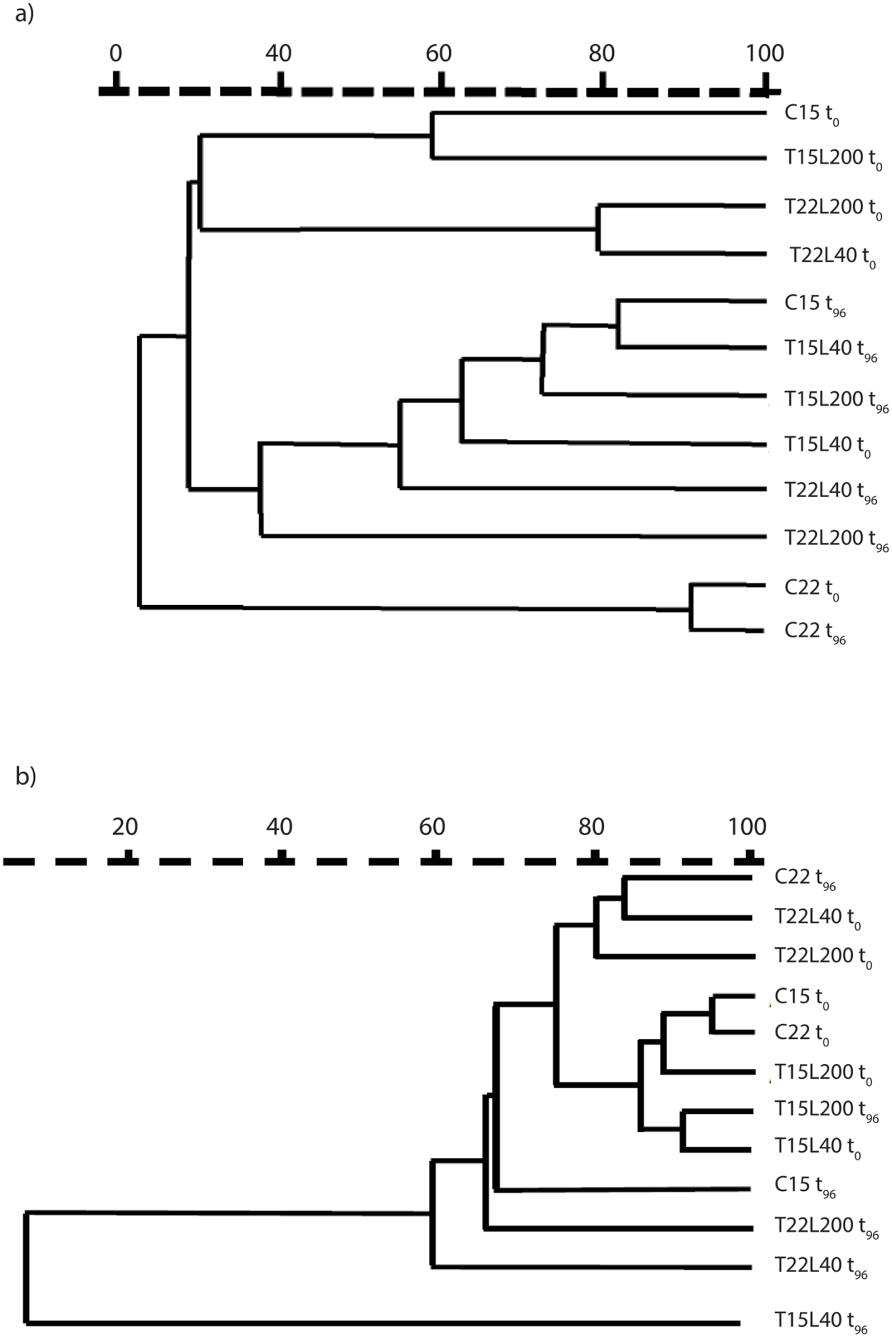
Similarity comparison of the bacterial (a) and archaeal (b) diversity between seawater, at 15°C (C15) and 22°C (C22), and the 4 experimental conditions (T15L40, T15L200, T22L40 and T22L200) just after the mucus was released (t_0_) and after 96 h of incubation (t_96_). The dendrograms were designed using GelCompar II software and they represent the clusters obtained from the 16S rRNA PCR-DGGE analyses.

DGGE produced distinct banding patterns for each sample with numerous discrete bands demonstrating the presence of diverse prokaryotic communities during the experiment. There was a significant effect of the experimental conditions and incubation time on the prokaryotic structure, as revealed by the changes observed in the number and intensity of bands detected for both the Eubacteria and the Archaea. From 6 to 20 bands were indeed observed for the Eubacteria and between 10 and 23 for the Archaea (Fig 7a and 7b), which presented, on average, a higher total number of bands per sample compared to bacteria. The number of bacterial bands decreased from *ca*. 20 to *ca*. 12 in the mucus maintained at 15°C and increased at 22°C with no variation in T22L200. Conversely, for the Archaea, the number of bands decreased during the incubation, from *ca*. 17 to 12 bands at 15°C and from *ca*. 17 to 15 bands at 22°C. The diversity was always higher at 15°C than at 22°C. The cluster analysis distinguished 3 bacterial and 4 archaeal patterns, with a maximum of *ca*. 20% of resemblance between the bacterial patterns and up to 75% for the archaeal ones (Fig 8a and 8b). These patterns highlighted differences in bacterial diversity between control seawater and mucus, suggesting that specific bacterial species are required to use the organic matter concentrated in the mucus. In addition, there was a clear difference in bacterial diversity between the beginning (T_0_) and after 96_h in the mucus maintained at 22°C. This difference was less evident in the mucus maintained at 15°C. There was a time effect for archaeal diversity, samples being generally different between the beginning and the end of the incubation.

## Discussion

Our study provides the first clues on OM fluxes in the temperate scleractinian coral *Cladocora caespitosa*, which forms large banks in the Mediterranean Sea. They point out the high nutritional value of the released OM, which is enriched in nitrogen, and mainly composed of dissolved material directly available for the growth of the surrounding micro-organisms. In addition, by simultaneously quantifying glucosidase and aminopeptidase enzymatic activities, and the growth of virus-like particles and prokaryotes in the OM released, this study allows a deeper understanding of the bacterial-mediated OM recycling and associated food webs. It has highlighted a rapid OM recycling and prokaryotes growth, but also a modification of the composition of heterotrophic bacteria, likely towards the most-efficient species. Finally, it has identified the optimal environmental conditions for a maximal OM release and recycling rate. All together, these results show that *C*. *caespitosa* appears to function as an important trophic pathway for organic matter from the benthic environment to the pelagic food chains in temperate environments with a multitude of impacts on microbial communities and biological interactions that remain to be studied. Temperature and light significantly changed the metabolism of *C*. *caespitosa* (Fig 1). Temperature was an important factor in the regulation of photosynthetic pigments and proteins. Chlorophyll concentrations per skeletal surface area or per symbiont cell were indeed 8 to 10 times lower at 22°C than at 15°C, and protein concentrations decreased by at least two times at 22°C (Fig 1a, 1b and 1c). This thermal effect can be considered more as an acclimation than a bleaching process [46], because symbionts presented the inverse trend, and reached their highest density at 22°C under high light (representing the summer condition). In this condition, symbionts density (10 to 12 x 10^6^ cells cm^-2^, Fig 1d) was 5 to 6 times higher than the mean concentration observed in tropical scleractinian corals [47, 48], and allowed *C*. *caespitosa* to optimize light capture, rates of photosynthesis and nutrient acquisition. This observation confirms previous *in situ* measurements showing that *C*. *caespitosa* reaches its maximal symbiont density and autotrophic carbon acquisition in spring-summer, when light is sufficient to sustain photosynthetic rates [49, 50]. Conversely, under low irradiance, and particularly under high temperature, symbiont density was reduced from 12 x 10^6^ to <2 x 10^6^ cells cm^-2^ (Fig 1d), indicating that symbionts did not receive enough light to optimise their photosynthetic rate and be profitable for the symbiotic association. This reduction is also an adaptive trait of this temperate symbiosis, which cannot shift symbiont genotype to adapt to environmental changes [51], since they have only access to two clades [52] instead of eight for tropical anthozoans [53, 54].

As for the holobiont metabolism, the nature and amount of OM produced by *C*. *caespitosa* depended on both temperature and light (Fig 2, Table 1). Whereas the percentage of PON to TON was the highest in the “summer condition” (T22L200, 45% of PON), the contribution of POC to TOC was the smallest (10%). This contribution increased to 50% under low light and temperature (“winter condition”, T15L40), either due to an increase in large prokaryotes colonizing coral OM (Fig 5), or differences in the nature of the photosynthates produced by the symbionts. Nonetheless, *C*. *caespitosa* released, in all conditions, more dissolved than particulate material (Fig 2, Table 1). This stands in contrast to most other tropical scleractinian corals [55] but is in agreement with observations made with other temperate coral species occurring in deep and cold environments [25]. The TOC:TON ratio was very low (ranging from 1.6 to 3.7) indicating that OM was mainly proteinaceous. Although low C:N ratios of OM released by corals was observed in previous studies [24, 56], and again in cold-water species [25], the ratio for *C*. *caespitosa* was lower, excepted in the T15L200 condition which was in range of the tropical scleractinian coral *Porites sp*. [24]. The amount of TOC produced by *C*. *caespitosa* (a maximum of 67 nmol h^-1^ cm^-2^ or 8.12 mg m^-2^ h^-1^, Fig 2a) was higher than the mean value reported for most tropical and deep sea scleractinian corals when using the same beaker-incubation technique (values ranging between 1 and 3 mg m^-2^ h^-1^, with a maximum at 7 mg m^-2^ h^-1^ for *Stylophora pistillata*, [24, 25, 57], but was lower than the first estimate for *C*. *caespitosa* by Herndl and Velimirov [27]. The same conclusion is reached with TON production by *C*. *caespitosa*, which was at least 10 times higher (2.29 mg m^-2^ h^-1^, Fig 2b) than the mean value of TON released by tropical scleractinian corals [24]. The high nitrogen content of *C*. *caespitosa* OM might be explained by the relatively rich environment, in which this coral species lives. In oligotrophic conditions, such as those prevailing in tropical reef waters, corals tend to retain nitrogen, considered as a limiting factor and tend to even take up DOC and DON for their own needs [24]. Conversely, *C*. *caespitosa* colonies used in this study originated from a eutrophicated environment, rich in organic and inorganic food, driven by one of the major flume of the area [28]. The important release rates of DON and PON by *C*. *caespitosa* highlight the non-limitation of this species by inorganic nutrients.

*C*. *caespitosa* OM has thus a very high nutritive value, because it is abundant, nitrogen enriched, and mainly composed of dissolved material directly usable by micro-organisms [58]. This particularity of *C*. *caespitosa* OM explains the high aminopeptidase activity observed, up to 578 fold the α-glucosidase activity in the freshly released OM (Fig 3). Aminopeptidase activity decreased within 4 h in all conditions, even under low temperature, because organic nitrogen was immediately degraded. This highlights the rapid use and important need of nitrogen for bacterial (and Archaeal) growth, which significantly increased in 24 h, by 1.25 fold at 15°C, and up to 13 fold at 22°C (Fig 6). This higher increase in bacterial growth at 22°C goes hand in hand with the higher aminopeptidase activity at this temperature. Growth rates reached values as high as 0.4 h^-1^, which corresponds to a production of 7.44 μg C L^-1^ d^-1^. This value is higher than the productivity monitored in the water column of the Mediterranean Sea, where production rates range from 0.78 to 1.16 μg C L^-1^ d^-1^ in the Aegean Sea [59] and a maximum of 3.6 μg C L^-1^ d^-1^ in the Ligurian Sea [60]. *C*. *caespitosa* mucus has thus a high nutritional value [61] (due to high nitrogen contents) for the temperate microbial food web. Nitrogen is known to limit bacterioplankton in many oceanic environments [62, 63], and is thus rapidly used by planktonic organisms when available.

Conversely to the organic nitrogen, which was recycled immediately, a maximum of 3% of the organic carbon released was recycled each hour (Fig 4). This percentage is at least twice lower than those estimated using bacterial respiration in experiments involving tropical corals [8, 64]. However, our measurements took only into account the amount degraded by the alpha-glucosidase and might have underestimated the degradation of carbon compounds by other enzymes, such as the ß-glucosidase. Overall, carbon was degraded much more slowly than nitrogen (Fig 3), suggesting that bacteria in the mucus were not carbon-limited, and the growth observed was more due to a nitrogen than a carbon enrichment of the seawater. Although nutrient limitation for prokaryote production is heterogeneous in the Mediterranean Sea and varies with season [65, 66], phosphorus and nitrogen represent the two main limiting factors all year round [66, 67]. The high bacterial production rates observed in the nitrogen-enriched OM produced by *C*. *caespitosa* tend to confirm this general limitation in nitrogen for Mediterranean bacteria.

Another difference of *C*. *caespitosa* OM compared to those of tropical species, in addition to its chemical composition rich in nitrogen and dissolved material also relies in the amount of heterotrophs colonizing it. Although the abundances of VLPs and prokaryotes in coral mucus depend on the coral species [68], heterotroph abundances were an order of magnitude lower than those measured *in-situ* right above corals, whereas the VLP abundance was comparable [69]. In other word, *C*. *caespitosa* OM was highly enriched in VLPs compared to other OMs, up to 5 fold those for tropical corals, and this was not associated with any visible disease of the colonies of *C*. *caespitosa* [69]. In addition, there was a close link between prokaryotic and VLP growth in the OM released by *C*. *caespitosa* (Fig 6). A remaining question to be solved in the bacterial-virus interactions is which factor, substrate supply (bottom-up) or viruses (top-down), plays the more dominant role in regulating bacterial production [70, 71]. In the coral mucus, based on VPR and bacterial abundances, high DOM concentrations seem to alleviate the viral lytic pressure on bacteria, which kept on growing during at least 72 h while viruses developed in parallel. The same pattern was observed in a recent study [71], with a lower bacterial mortality by viruses in eutrophic than oligotrophic waters, because high DOC loading in eutrophic waters improved bacterial metabolic activity by alleviating virus-induced mortality of bacteria.

Mucus of *C*. *caespitosa* appears as a perfect medium to enhance the microbial food web *via* a rapid bacterial growth. However, it also induces changes in the composition of the microbial communities. Indeed, although the quick and cost-effective PCR-DGGE technique captures only a small fraction of the diversity, and target only the dominant groups, we observed that coral OM induced a bacterial selection as previously noticed with other coral OM [7]. Interestingly, this study also highlights a strong influence of the abiotic factors (merely the temperature) on this selection, which should be investigated in further and more detailed studies, especially in a context of climate warming. Nevertheless, the selection does not seem to be applied on Archaea, which presented a high similarity between the control seawater and the different conditions at the beginning of the incubation (Fig 8b). It confirms previous observations performed with tropical coral OM (review by Rosenberg et al [72]). The diversity of Archaea decreased in *C*. *caespitosa* OM after 96 h, suggesting that this group of prokaryotes were not the most efficient in degrading and using such OM, although there are known to be good nutrient recyclers [73, 74] and likely here to play a key role in N cycling.

## Conclusion

These comprehensive data provide an important basis for the understanding of organic matter dynamics in temperate waters where corals are dominant. In all conditions, coral colonies released DOM and POM into the surrounding seawater, thereby providing a source of energy, rich in carbon and nitrogen compounds, to the planktonic communities. The released OM was predominantly composed of DOC and DON, highly stimulating microbial growth and production, as well as the abundances of VLP. It remains a potential target issue of future studies to know if this enhancement of the microbial loop in the vicinity of *C*. *caespitosa* may help this species, whose nutrition is based on heterotrophy in winter, to prey on microbes. Nevertheless, OM release acts as a trophic link between corals and the planktonic organisms in the Mediterranean Sea.

## Supporting Information

S1 Table Minimal dataset. (XLSX)
